# Neuroprotective Therapies after Perinatal Hypoxic-Ischemic Brain Injury

**DOI:** 10.3390/brainsci3010191

**Published:** 2013-03-05

**Authors:** Felipe Goñi de Cerio, Idoia Lara-Celador, Antonia Alvarez, Enrique Hilario

**Affiliations:** 1 Biotechnology Area, GAIKER Technology Centre, Parque Tecnológico de Zamudio Ed 202, 48170 Zamudio, Vizcaya, Spain; E-Mail: goni@gaiker.es; 2 Department of Cell Biology and Histology, School of Medicine and Dentistry, University of the Basque Country, 48949 Leioa, Bizkaia, Spain; E-Mails: poliacrilamida@hotmail.com (I.L.-C.); antoniaangeles.alvarez@ehu.es (A.A.)

**Keywords:** perinatal hypoxia-ischemia (HI), brain injury, neuroprotective strategies

## Abstract

Hypoxic-ischemic (HI) brain injury is one of the main causes of disabilities in term-born infants. It is the result of a deprivation of oxygen and glucose in the neural tissue. As one of the most important causes of brain damage in the newborn period, the neonatal HI event is a devastating condition that can lead to long-term neurological deficits or even death. The pattern of this injury occurs in two phases, the first one is a primary energy failure related to the HI event and the second phase is an energy failure that takes place some hours later. Injuries that occur in response to these events are often manifested as severe cognitive and motor disturbances over time. Due to difficulties regarding the early diagnosis and treatment of HI injury, there is an increasing need to find effective therapies as new opportunities for the reduction of brain damage and its long term effects. Some of these therapies are focused on prevention of the production of reactive oxygen species, anti-inflammatory effects, anti-apoptotic interventions and in a later stage, the stimulation of neurotrophic properties in the neonatal brain which could be targeted to promote neuronal and oligodendrocyte regeneration.

## 1. Introduction

Hypoxic-ischemic (HI) encephalopathy is one of the major causes of disability and death in newborn infants worldwide [1]. An estimated four million babies die every year during the neonatal period, and one quarter of these deaths are attributed to HI [2]. Neonatal encephalopathy is a common clinical condition affecting approximately 2 in 1000 neonates [3], and accounts for a substantial proportion of admissions to neonatal intensive care; 10%–15% of cases will die in the neonatal unit, 10%–15% will develop cerebral palsy and up to 40% will have other significant disabilities including blindness, deafness, autism, epilepsy, global developmental delay, as well as problems with cognition, memory, fine motor skills and behavior [4,5,6,7,8]. These problems are observed throughout development with a tremendous impact on the affected child, its family and society [9,10]. 

Despite important progress in obstetric and neonatal care during the last decades, perinatal HI is still one of the most important causes of neonatal brain injury and its associated adverse developmental outcome [8,11,12]. The severity, intensity and timing of asphyxia, as well as a selective ischemic vulnerability and the immaturity of the brain, determine the extension and the degree of severity of the ensuing damage and long-term neurodevelopmental impairment [13,14,15,16].

Neuropathological studies indicate that many critical neuronal groups are more vulnerable to HI injury in newborns (immature brain) than in adults, particularly related to enhanced density and function of excitatory amino acid receptors as well as enhanced vulnerability to attack by reactive oxygen species (ROS) and reactive nitrogen species [17]. In fact, the immature brain has more blood vessels, higher water content, lower myelin, a poorly developed cortex, and a more prominent germinal matrix than the mature brain [10]. These characteristics make the preterm brain more susceptible to HI damage. 

Due to these immature brain characteristics, it is necessary to focus on the period of time following the HI event, when the therapeutic strategies could be efficacious in the reduction of brain damage to improve the care in perinatal HI. This period is normally short and may vary from 2 to 6 h; therefore, a rapid identification could facilitate the application of diverse rescue strategies. In order to reduce neurological consequences derived from HI injury, it is necessary to improve some actions, such as monitoring the perinatal period [18,19].

## 2. Pathogenesis of Perinatal Brain

The principal pathogenic mechanism underlying neurological damage resulting from HI is the deprivation of the glucose and oxygen supply, which causes a primary energy failure and initiates a cascade of biochemical events leading to cell dysfunction and ultimately to cell death [20,21]. 

**Figure 1:**
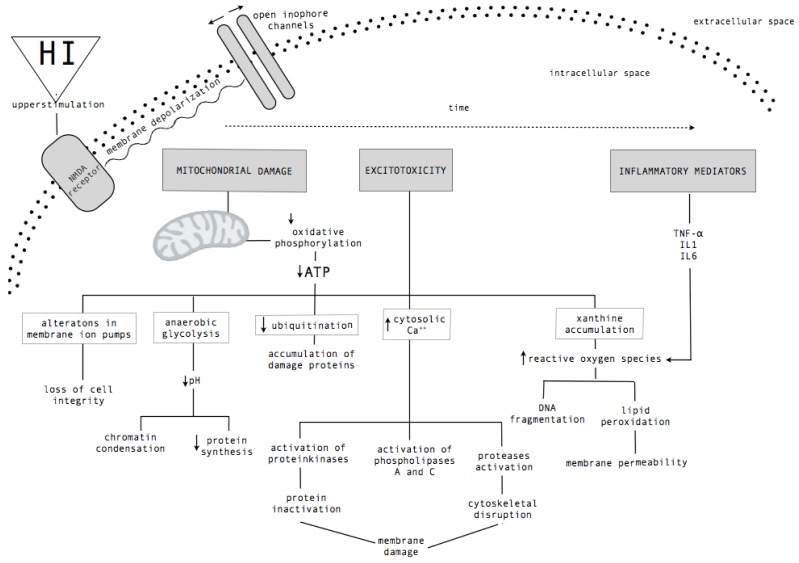
Cascade of biochemical mechanism after hypoxic-ischemic (HI) brain injury. A schematic diagram that summarizes the cellular and molecular events triggered after HI injury in the developing brain. Mitochondrial damage, the cytotoxic levels of intracellular calcium and the release of inflammatory mediators cause metabolic failure, oxidative stress and ultimately the cell death.

Brain damage following a perinatal HI is an evolving process, which is comprised of two phases [13]. A first phase consists of an early energetic failure, where the oxidative energy metabolism of cells decreases and it promotes necrotic death. This is followed by a second phase of cell death, a late energetic failure, which occurs during reperfusion and reoxygenation several hours after the initial event and lasts for days [10,22,23]. The pathophysiology of this late energetic failure initiates a cascade of biochemical events (Figure 1), which involve nitric oxide synthases activation, the production of cytotoxic free radicals, inflammation, membrane dysfunction and apoptosis, among others [24]. 

## 3. Calcium Influx and Free Radical Formation

During the late energetic failure, a consequent reperfusion injury often deteriorates the brain metabolism by increasing the oxidative stress damage. Particular roles for increase in extracellular glutamate, excessive activation of glutamate receptors (excitotoxicity), increase in cytosolic calcium (Ca^2+^) and generation of free radicals are emphasized [12,25,26,27]. 

Loss of mitochondrial membrane potential, combined with high concentrations of glutamate, opens calcium-permeable NMDA glutamate channels and voltage-gated calcium channels allowing calcium to move into neurons [28]. This fact triggers enhanced production of free radicals and activation of lipases, proteases, and endonucleases. 

As a consequence of lipases and proteases activation, the release of free fatty acids, especially arachidonic acid, will activate cyclooxygenase and will catalyze the formation of prostaglandins, which will liberate super-oxide free radicals. In addition, the formation of oxygen free radicals is also enhanced via hypoxanthine metabolization. Hypoxanthine is formed during the HI and metabolized to uric acid. Collectively, these processes will lead to a surge of the superoxide free radicals, which play a central role in further production of free radicals and other toxic compounds [12,25,26,27].

The prominence of an NMDA-mediated injury in the immature brain is related to the fact that NMDA receptors are functionally upregulated in the perinatal period due to their role in activity-dependent neuronal plasticity [29]. Immature NMDA channels open more easily and stay open longer than adult channels, and the voltage-dependent magnesium block that is normally present in adult channels at resting membrane potentials is more easily relieved in the perinatal period [30].

## 4. Nitric Oxide Synthases Activation

Open NMDA channels allow calcium to enter into the intracellular compartment and activate neuronal nitric oxide synthase (nNOS), leading to production of the oxygen free radical nitric oxide (NO) [31,32]. Then, NO can react with superoxide to form toxic peroxynitrite, which can add nitrate to tyrosine groups on proteins. This reaction contributes to the production of hydroxyl radicals, causing lipid peroxidation of proteins and DNA, which produce to further damage to brain tissue [33,34,35,36]. NO can also disrupt mitochondrial respiration by impairing the function of cytochrome oxidase from complex 4 and complex 1, which increases the production of superoxide and peroxynitrite ions in mitochondria, especially during hypoxia [32,37].

## 5. Inflammation

Inflammation plays an important part in the excite-oxidative cascade of injury in the perinatal period [38]. Three to twelve hours after reperfusion and reoxygenation an inflammatory response, which is probably induced by excessive free radical production and high levels of extracellular glutamate, pro- and anti-inflammatory cytokines such as TNF-α, IL-1, IL-6, IL-8 and IL-10 will be activated [39]. 

Likewise, the activation of two transcription factors, Nuclear Factor kappa B (NF-κB) and c-Jun *N*-terminal kinase (JNK), play a central role in the post-HI inflammatory process. In addition, these transcription factors can regulate expression of pro- and anti-apoptotic proteins and thus can contribute to damage or neuroprotection [40,41,42].

## 6. Apoptosis Activation

Apoptotic activity contributes to brain damage in the neonate and is an important pathway in the process of delayed neuronal death. Apoptosis is an energy-dependent process and ATP is required for apoptosome formation and subsequent caspase activation [43,44]. Caspases and especially the caspase-3 are activated in this process and bring about most of the changes that characterize apoptotic cell death [45]. Activated caspase-3 is expressed at higher levels in the developing brain after perinatal HI, giving rise to the assumption that apoptotic mechanisms of neuronal cell death seem to be more important in neonatal brain injury than adults [46]. Increased knowledge about the factors that determine when or how cells die after HI is important since it might enable salvage tissue through use of drugs, growth factors or treatment interventions that influence brain activity [47,48].

## 7. Neuroprotective Therapies

**Figure 2:**
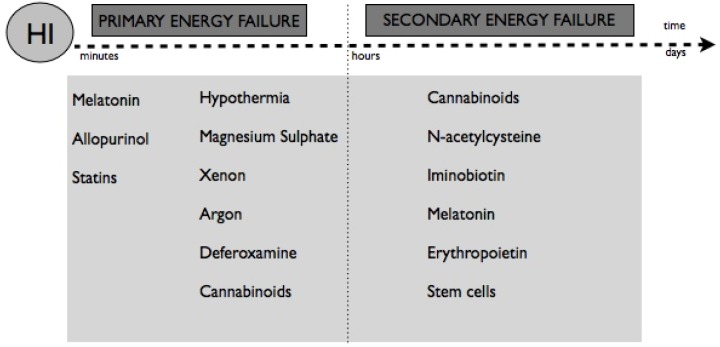
Neuroprotective therapies and their optimal moment of administration, according to their mechanisms of action. In this sense, pharmacological antioxidant therapies such as melatonin, allopurinol, hypothermia or magnesium sulfate could be useful just after the HI event. After the reperfusion, when the secondary energy failure takes place, other therapeutic options like cannabinoids, erythropoietin or iminobiotin, which have anti-inflammation and anti-apoptotic effects, could be promising therapies.

Many potential neuroprotective therapies that target specific pathways in the pathophysiology of HI brain injury have been investigated. At present, no individual neuroprotective agents have been proven safe and effective against neurological sequels after HI events in neonates. The insight into the biochemical and cellular mechanisms of neuronal injury after HI helps to provide interventions to interrupt those deleterious cascades derived from the event [49]. Pharmacological and non pharmacological therapies should start at different points of time after the HI event, in their optimal therapeutic window, according to their mechanisms of action (Figure 2). Moreover, some of these therapies are supplied pre-HI event. Anyway, the goals of these therapies are: reduce cerebral damage by decreasing the formation of toxic free-radicals, inhibit the excessive influx of calcium into neurons and minimize cerebral edema principally [50,51].

Likewise, to improve the care in perinatal HI, it is necessary to focus on the period of time following HI event, when the therapeutic strategies could be efficacious in the reduction of brain damage. This period is normally short and may vary from 2 to 6 h and therefore a rapid identification would facilitate the application of diverse rescue strategies. 

In these sense, up to the present moment, some of the most useful therapies have been appeared, such as *N*-acetylcysteine and allopurinol, magnesium sulfate, glutamate receptor blockers, erythropoietin and hypothermia [52]. These pharmacological and non-pharmacological interventions progress to minimize the extent of damage along the evolving process after HI brain injury [53,54,55,56,57,58].

## 8. Non-Pharmacological Therapies

Among the nonpharmacologic therapies for the treatment of brain injury, hypercapnea and hypothermia stand out. On the one hand, in experimental assays with rats, hypercapnea has been reported to reduce lung injury, increase cerebral blood flow, and protect the immature brain from HI injury [59]. On the other hand, hypothermia appears to be the most reliable intervention available at the moment for reducing the risk of death or disability in infants with brain injury [60,61]. Multiple animal experimental models have demonstrated that a “mild-moderate” reduction in brain temperature of 2–3 °C below normal is beneficial when utilized during HI, during resuscitation and after the event to prevent or attenuate neuropathological damage. The temperature reduction in a moderate grade (32–34 °C) has now become standard of care for neonatal HI brain injury. For each 1°C decrease in core temperature, the cerebral metabolic rate decreases by 6% to 7% [62,63]. 

Up to date, information derived from magnetic resonance imaging studies related to hypothermia therapy suggests that head and total body cooling are associated with a decrease of basal ganglia/thalamic brain lesions incidence [64]. The mechanisms based on the hypothermic neuroprotection are the increase of neuronal survival in the basal ganglia and the suppression of caspase-3 activation [65]. Hypothermia has also been shown to suppress microglial activation [66]. Furthermore, the inflammation and expression of TNF-α, IL-1β and IL-18 are reduced [67] whereas there is an increase of the anti-inflammatory cytokine IL-10 [66,68]. At a cellular level, hypothermia protects the cell wall and maintains the integrity of the lipoprotein membrane [69]. Furthermore, it decreases enzymatic reactions that lead to cell damage or death [70,71]. In addition, hypothermia inhibits activation of NMDA receptors [72]. At tissue level, hypothermia improves oxygen supply to areas of ischemic brain and decreases intracranial pressure [73].

Several trials of hypothermia in human newborns have been performed during last decades, applying two different methods: whole-body cooling and selective head cooling. Although neither method has been demonstrated to be superior, each mode of cooling has unique properties. These larger clinical trials in human newborns described reproducible approaches to hypothermic therapies and confirmed the feasibility of such therapies [74,75,76,77]. In many of the experienced centers involved in the multicenter trials, hypothermia is becoming “standard care” [78]. 

The benefit of hypothermia in reducing death and major disability in the survivors has been confirmed, but a number of important questions remain [79]. The optimal depth of cooling has yet to be determined in asphyxiated infants. Similarly, optimal duration remains unclear. The optimal mode of delivery—selective head *vs.* whole-body cooling—has not been studied [80]. As well as the exact duration of the therapeutic window in humans is unknown and likely related to inflammatory influences, nutrition, brain maturation and genetic predisposition [81]. Successful dissemination of this new therapy will require improved identification of infants with perinatal HI and the creation of systems that can institute therapy in a timely manner.

## 9. Pharmacological Therapies

### 9.1. Therapeutical Strategies Related to Antioxidants

Recent studies using a variety of pharmacological agents have noted that their administration followed by perinatal HI could contribute to effectiveness. In this sense, the main point of using these specific drugs is focused on reducing the toxic free radicals and inhibiting the excessive influx of calcium into neurons to minimize cerebral oedema caused by HI [9,52,82].

Regarding free radical formation after the HI event, allupurinol could reduce the formation of free-radicals that cause tissue damage and could help to maintain the blood-brain barrier. Allopurinol and its metabolite oxypurinol are inhibitors of xanthine oxidase, the enzyme involved in superoxide production, especially during reperfusion damage [83]. The difference between both of them is that oxypurinol crosses the blood brain barrier more easily than allopurinol.

Neuroprotective effects of allopurinol administered after the event had been observed in seven-day-old rats [84] and in newborn lambs [85]. Reactive oxygen species induced after brain cell injury can be reduced through inhibition of xanthine oxidase, present in capillary endothelial cells [86], by allopurinol and oxypurinol [87]. Other neuroprotective pathways of allopurinol are the direct scavenging of free radicals demonstrated *in vitro* with high concentrations of allopurinol [88], inhibition of neutrophil accumulation [89], chelation of metal ions such as ferric iron [90] and facilitation of electron transport from ferrous iron to ferric cytochrome C [91].

A recent human pilot study has shown promising results when administrated immediately prior to delivery when suspecting fetal asphyxia. Five hundred mg of allopurinol or placebo was administrated intravenously to 53 pregnant women in labor (54 fetuses with a gestational age >36 weeks and signs of fetal hypoxia). It proved a reduction of biomarkers of neuronal damage [92]. However, it is possible that allopurinol has no positive effect when started too late and at low doses [93].

Other possible candidates, which are widely prescribed to lower cholesterol in hyperlipidemic patients at risk of cardiovascular diseases, are statins (3-hydroxy-3-methylglutaryl coenzyme A reductase inhibitors). Experimental evidence suggests that statins also possess properties that may confer to this class of drugs a prophylactic neuroprotective effect in stroke [94]. Although human treatment with statins seems still far away, Neuroprotection was associated with reduction of cytokine expression, caspase-3 activation and apoptotic cell death. However, according to research on immature rats, the neuroprotective effect was not associated with changes in eNOS expression [95]. Perinatal neuroprotection was observed using a prophylactic, high dose of statin (20 mg/kg), administered for seven days before the onset of ischemia [96] but not noticed when the drug was administered after the event [95]. In addition, other study reported the prophylactically administration of Simvastatin attenuated the HI-induced oligodendrocytes injury, inhibited microglial activation and reduced the numbers of pyknotic cells and neuronal loss [97]. However, the molecular mechanism by which the neuroprotective effect is achieved is not fully understood.

Likewise, up to the moment, different noble gases have been studied as new neuroprotection therapies. On one hand, xenon, a non-competitive antagonist of the *N*-methyl-d-aspartate (NMDA) subtype of the glutamate receptor [98], appears to be superior to other NMDA antagonists because it has additional mechanisms of action, such as the inhibition of AMPA and kainate receptors and the reduction of neurotransmitter release [99]. Other actions of xenon include inhibition of the calcium/calmodulin dependent protein kinase II [100], activation of anti-apoptotic effectors Bcl-XL and Bcl-2 [101] and induced expression of hypoxia inducible factor 1α [102]. Xenon is neuroprotective following HI in neonatal rats [103,104] and is effective even when its administration is delayed for some hours [105,106]. Moreover, the combination of xenon with hypothermia caused an effect, even at low concentrations or mild temperature reductions, while it supplied separately had no effect at all [103]. The major disadvantage of this intervention is that xenon is very expensive and its administration is rather complicated, since it requires intubation and ventilation of the patient, as well as a high percentage of xenon [12]. On the other hand, argon is a noble gas that, in contrast to xenon, is ubiquitous, cheap and widely applicable. Ryang *et al.* reported the argon neuroprotective role in an *in vivo* rat model of acute focal cerebral ischemia showing a significantly reduction of infarct volumes and better functional outcomes. However, other studies have pointed out the absence of a therapeutic effect, no advantage in acute survival 24 h after transient middle cerebral artery occlusion was demonstrated [107]

Furthermore, administration of magnesium sulfate (MgSO_4_) has been suggested to act as a neuroprotective agent. MgSO_4_is an NMDA receptor antagonist, which prevents excitotoxic calcium-induced injury through the non-competitive voltage-dependent inhibition of NMDA receptor. This inhibition reduces calcium entry into the cell [108,109,110,111]. Magnesium sulfate may also have direct actions on mitochondrial activity, anticonvulsant properties and haemodynamic effects by increasing cerebral blood flow. Moreover, animal data suggest that MgSO_4_ may serve an antiapoptotic role and prevent neuronal cell loss [112,113,114]. An additive effect in reduction of the infarct area, when magnesium sulfate is associated to mild hypothermia, has been observed in rats [115]. 

Nowadays, there is no general consensus about the value of magnesium as a neuroprotective agent. Previous reports suggested that MgSO_4_ administration prevented the effects of energy depletion after an HI event in newborn children trials [116] and altered important enzymes in erythrocyte membrane from asphyxiated newborns, reducing the postasphyxial damage [117]. However, other multicenter trials have pointed out on the one hand the absence of a therapeutic effect [118,119] and on the other hand, magnesium administration has even been considered to be harmful for the fetus [120,121], although this opinion is not unanimously held [122,123,124] and the question is still unclear. These paradoxical perspectives regarding the neuroprotective effect of MgSO_4_ administration could be the consequence of the variability in the study design, depending on the dose and the experimental model, making it difficult to compare the outcomes directly. Studies with newborn rodents and different magnesium doses presented divergent results, including neuroprotection [125,126,127,128,129,130] or its absence [115,131,132,133,134,135]. On the other hand, although lamb or pig models are closer to humans [136], up to date there are few studies on the protective effect of MgSO_4_ administration in these newborn mammals suffering neonatal HI encephalopathy [114].

In the last years, several studies have pointed out cannabinoids as substances with high potential as neuroprotective compounds, both in acute neurodegenerative diseases, as HI or traumatic brain damage and in chronic processes as multiple sclerosis, Parkinson’s disease and Alzheimer’s disease [137,138,139]. These substances have emerged as neuroprotectants due to the fact that can modulate neuronal and glial response. Besides, cannabinoids have endothelial cell functions, anti-excitotoxic [140,141] anti-inflammatory [142,143] and vasodilator effects [144], regulating at the same time the calcium homeostasis [145,146]. 

Activation of cannabinoid receptors induces the closure of Ca^2+^ channels, consequently inducing a neuroprotection through the reduction of glutamate release [147,148]. Drugs reducing glutamate release are of particular value according to neuroprotection in neonatal HI event, as glutamate receptor blockers are neurotoxic in immature brains [149]. In addition, cannabinoids reduce direct NMDA toxicity by downstream inhibition of Protein Kinase A signaling and NO generation [150]. 

Several *in vitro* studies have reported neuroprotective effects of cannabinoids related to their antioxidant effect [151,152]. In addition, *in vivo* models of neurodegenerative diseases have demonstrated antioxidant-related neuroprotective actions for cannabinoids [153]. Cannabinoids possess some other properties that account for their neuroprotective effects after a HI event: they are brain vasodilators [154,155], stabilize the blood-brain barrier and are involved in neuroproliferative processes [156]. Cannabinoids enhance energy metabolism of astrocytes [157] and protect these glial cells against cytotoxic and proapoptotic stimuli [158].

Administration of endogenous cannabinoids emerges as a novelty neuroprotective therapy due to the particularity that these substances take part on the natural mechanism for controlling damage. According to their neuroprotective effects, experimental *in vitro* studies confirmed that the endogenous cannabinoids AEA and 2-AG may attenuate the injury in cortical cells in an oxygen glucose deprivation model [159]. Taking into consideration an *in vivo* model of induced excitotoxicity endocannabinoid AEA protects the neuronal injury [160]. Moreover, according to closed head injury in mice, the administration of 2AG promotes significant reduction of brain oedema, better clinical recovery, reduced infarct volume and reduced hippocampal cell death [161]. Finally, the administration of these two different endocannabinoids after HI injury in perinatal rat model creates a remarkable amelioration of brain injury, reducing apoptotic cell death and contributing to the maintenance of mitochondrial functionality, as well as improving cellular parameters such as the influx of calcium and ROS production [162].

Among the anti-oxidant interventions for the treatment of perinatal brain injury, the melatonin is also a well focused possible therapy. Melatonin is an endogenously produced indoleamine that is primarily formed by the pineal gland. Melatonin has the ability to cross all morphophysiological barriers and therefore is distributed widely in tissues, cells and subcellular compartments including the brain. Various studies reported that melatonin might act as a neuroprotective agent in neonatal HI [163,164] and acts as a potent endogenous antioxidant by scavenging free radicals and upregulating antioxidant pathways. The activity and expression of antioxidant enzymes such as superoxide dismutase, glutathione catalase, glutathione peroxidase and glutathione reductase have been shown to be increased by melatonin, supporting its indirect antioxidant action. Further evidence of the antioxidant effect of melatonin is provided by its ability to reduce lipid peroxidation, a degradative phenomenon involved in the pathogenesis of many diseases [165].

Another alternative is the use of antioxidants such as erythropoietin, which has antiapoptotic and angiogenic properties [166] and provides neuroprotection and neurogenesis in neonatal rats [167,168]. Vitamin E is also hypothesized as an antioxidant and free-radical scavenger to be effective reducing the risk and severity of HI damage [169]. Moreover, deferoxamine prevents the formation of free radicals from iron since it is a free metal-ion chelator. Deferoxamine reduces the severity of brain injury and improves cerebral metabolism in animal models of HI when supplied during reperfusion [170]. However, some toxic effects have been detected when administrated at high dose in preterm baboons [171].

### 9.2. Therapeutical Strategies Related to Anti-Inflammation and Anti-Apoptosis

Apart from producing an antioxidant effect, cannabinoids can also play a key role in peripheral and brain immune functions, including the inhibition of the inflammatory mediators release, such as nitric oxide, interleukin-2 and TNF-α, the inhibition of the cell-mediated immune processes activation and the inhibition of proliferation and chemotaxis [172,173].

Moreover, some authors have indicated that cannabinoid WIN55212 reduces apoptotic cell death through the maintenance of mitochondrial integrity and functionality in all regions studied [174], it promotes neurogenesis in subventricular zone, oligodendrogenesis, white matter remyelination and neuroblast generation after neonatal HI event [175]. Besides, the CB1 antagonist AM281 and the DAG-lipase inhibitor O-3640, exacerbates the detrimental effects in an oxygen glucose deprivation *in vitro* model by releasing glutamate in excess. The CB2 receptor agonist, 0-1966, has been found to increase blood flow to the brain and therefore attenuates neuroinflammation in an animal model of stroke [159]. These data support the hypothesis that the protective effects of cannabinoids derive from its anti-apoptotic and anti-inflammatory effects, opening a new gate about its possible use as neuroprotective targets after perinatal HI.

Regarding to anti-inflammatory effect after the HI event, *N*-acetylcysteine (NAC) has been used in some pilot studies. NAC is a free radical scavenger and restores intracellular glutathione levels, attenuating reperfusion injury, decreasing inflammation and NO production in models of stroke [176]. Besides, it has low toxicity and it is able to cross the placenta and blood-brain barrier. In a clinical trial, extremely low birth weight newborns received NAC by continuous infusion during the first six days of life in order to reduce chronic lung disease incidence [177]. When combined with hypothermia, NAC decreased infarct volume, improved myelin expression and functional outcomes after focal HI injury in seven-day-old rats exposed to 2 h of carotid ligation and hypoxia [178].

Furthermore, melatonin also has antiapoptotic and anti-inflammatory effects. It prevents the translocation of NF-κB to the nucleus, therefore reduces the up-regulation of pro-inflammatory cytokines [179] and it reduces the expression of pro-inflammatory genes such as cyclooxygenase-2 (COX2) and iNOS [180]. Welin *et al.* demonstrated that post-asphyxia melatonin treatment attenuated the increase in activated microglia and 8-isoprostane (a marker of lipid peroxidation) production and, at the same time, reduced the number of apoptotic cells in the cerebral white matter in midge station fetal sheep [181]. It may act at different levels by decreasing inflammation with some of the multiple mechanisms responsible for the progression of the neurodegenerative process. Therefore, melatonin may represent a promising neuroprotectant, on its own or as an additional adjunctive therapy, for reducing brain injury and its long-term sequaelae in infants [181,182].

Recent studies using erythropoietin (EPO) have noted that its administration following perinatal HI could contribute to effectiveness. The EPO, which was originally identified for its role in erythropoiesis, was found to play a variety of roles in modulation of the inflammatory response and has vasogenic effects [11]. It may activate antioxidant enzymes, decrease excitotoxic damage, induce anti-apoptotic and anti-inflammatory factors and inhibit lipid peroxidation [183]. Moreover, EPO regulates the balance of antiapoptotic and proapoptotic genes expression, increasing anti-apoptotic gene Bcl-2 levels [184].

EPO prevents the secondary delayed rise in IL-1β, attenuates the infiltration of leukocytes into the ipsilateral hemisphere [185] as well as the pro-inflammatory response in brain injured pups [186]. Neuroprotection with EPO has been documented in spinal cord injury, traumatic brain injury, ischemic stroke, and perinatal HI [187]. Administration of EPO, after HI event, promotes oligodendrogenesis leading to attenuated white matter injury concurrently with increased neurogenesis [188]. However, the mechanisms of EPO in different kinds of neural injury have not been clearly clarified, especially for neonatal brain injury.

Another neuroprotective strategy is the iminobiotin. Iminobiotin, an analog of biotin, has inhibited both nNOS and iNOS in experimental studies, so it could be considered as a neuroprotectant. Otherwise, *in vivo*, it provides long and short-term neuroprotection probably inhibiting cytochrome c-caspase 3, consequently hindering apoptotic pathways. Remarkably, only female rats were protected against brain injury, what suggests a gender specific effect [189].

In the last years, several studies have pointed to these candidates as substances with high potential as neuroprotective compounds, both in acute neurodegenerative diseases and in chronic processes. These pharmacological and non-pharmacological interventions are progressing to minimize the extent of damage along the evolving process after HI brain injury by modulating the neuronal response, anti-excitotoxic, anti-inflammatory, anti-apoptotic, vasodilatory effects as well as by regulating the calcium homeostasis.

## 10. Delayed Possibilities: Regeneration

During HI brain injury, neurons, glia and endothelial cells are damaged, thus reducing their functionality or dying. Endogenous regeneration mechanisms have been shown to exist in the brain with ischemic injury, stimulating neural stem cell proliferation and differentiation in cerebral neurogenic areas [190,191,192]. However, the capacity of the neonatal brain to respond to enhanced endogenous neurogenesis following neonatal HI may depend on timing and severity of event. In addition, endogenous neurogenesis may only partially restore brain damage after an HI event.

Recent advances in regenerative medicine suggest that stem cell transplantation may improve repair of the damaged brain [193]. Neural stem cells can renew and differentiate themselves between cells of all glial and neuronal lineages and populate the developing or the degenerating central nervous system regions.

Recent evidences suggest that HI induced brain damage can also be treated with mesenchymal stem cells (MSCs) [194]. MSCs may also secrete several trophic factors including colony stimulating factor-1, VEGF, basic fibroblast growth factor, nerve growth factor and brain derived neurotrophic factor [195]. In these sense, the intracranial administration of MSCs several days after HI event has shown a decreased histological damage and an improved outcome in rat HI model [196]. 

Stem cell transplantation has the potential to become a future neuroprotective and regenerative therapy for ischemic brain damage, however there are still hurdles to overcome before clinical application of stem cell transplantation can safely be considered.
